# Use of Information and Communication Technology Strategies to Increase Vaccination Coverage in Older Adults: A Systematic Review

**DOI:** 10.3390/vaccines11071274

**Published:** 2023-07-21

**Authors:** Alessandra Buja, Giulia Grotto, Mustapha Taha, Silvia Cocchio, Vincenzo Baldo

**Affiliations:** Department of Cardiac, Thoracic, Vascular Sciences, and Public Health, University of Padua, 35131 Padua, Italy

**Keywords:** older adults, information and communication technology, vaccine promotion, immunization, vaccination strategies

## Abstract

Background: Coverage rates of routinely recommended vaccines in older adults still fall below the targets established by international and national advisory committees. As a result, related diseases still have a high incidence, morbidity, and mortality. Information and Communication Technologies (ICT) could provide useful tools to improve immunization rates by bringing information directly to the target user at a relatively low cost. The present research aims to systematically review recent literature on interventions applying ICT to improve the uptake of influenza, pneumococcal, COVID-19 and herpes zoster immunization rates among older adults. Methods: Studies published in English between 1 January 2000 and 10 November 2022 were identified by searching electronic medical databases (PubMed, Scopus) and were independently reviewed by two different authors. A total of 22 studies were included in this review. Findings: Interventions applied the following ICT tools: phone calls, text messages, messages sent via personal electronic medical records, automated phone calls, remote patient monitoring in a home telehealth program and emails. In terms of the vaccines promoted, 11 studies prompted the influenza vaccine, four prompted the influenza and pneumococcal vaccines, three the pneumococcal vaccine, two the herpes zoster vaccine, one the COVID-19 vaccine and one both the pneumococcal and herpes zoster vaccines. Overall, more than half of the studies (n = 12) found some level of effectiveness of these ICT strategies in increasing vaccination rates among older adults, while five studies were partially effective (for specific vaccines or population subgroups), and five reported no significant effect. Conclusions: Prevention programs using ICT tools could be effective in promoting immunizations among older adults.

## 1. Introduction

Increasing and maintaining vaccination coverage is of fundamental importance to further decreasing the burden of vaccine-preventable diseases and thus has been identified as a public health priority [1,2]. Despite recommendations, immunization rates among individuals aged 65 years and older are generally low. Estimates of vaccination coverage in the United States in 2019 for three key adult vaccines—influenza, pneumococcal disease, and herpes zoster (HZ)—ranged from 41.2% for the HZ vaccine to 70.5% for the influenza vaccine [3]. Similarly, vaccination rates among the elderly in other high-income countries remain insufficient despite the availability of vaccines and despite convincing evidence-based medical and economic rationales. For example, data from the 2016–2017 influenza season showed that influenza vaccination coverage rates in European countries ranged from 2% to 72.8%, with a median of 47.1%; the highest vaccination coverage rates were recorded by the UK, which almost reached the European target of 75% [4]. European pneumococcal vaccination coverage among older adults is even lower, reaching 24.2% [5].

As a consequence, the burden of vaccine-preventable diseases remains high in older adults, contributing to substantial morbidity, mortality, and healthcare resource use and costs [6,7,8]. Older adults, together with people with chronic diseases (e.g., cancer patients), represent the population subgroup that would benefit most from vaccination, as they are at risk of developing serious complications. It has been estimated that between 70% and 85% of recent seasonal flu-related deaths have occurred in people 65 years and older, and that 50% to 70% of seasonal flu-related hospitalizations have also occurred among people in this age group [9].

Suboptimal vaccination coverage results from a variety of challenges and obstacles, not just the spread of vaccination hesitancy in the general population and the increased hesitancy of providers to vaccinate themselves and to recommend vaccination for their patients [10,11], but also from a lack of communication. Indeed, factors often associated with non-adoption of vaccination are the absence of awareness of the need for and importance of vaccination and the lack of vaccine recommendation by healthcare professionals [10]; the key points for effective immunization programs in the elderly are all related to patient contact and communication: improving access to vaccination, reminding patients of vaccination appointments, and increasing patient awareness of the importance of vaccination [12].

Immunization campaigns can be supported by different sources of Information and Communication Technologies (ICT) that can potentially bring information directly to the individuals. Although there is no single, universal definition of ICT, the term is generally accepted to refer to all devices, network components, applications and systems that, combined, enable people and organizations to interact in the digital world. ICT encompasses both the interconnected and mobile spheres powered by wireless networks. It also includes antiquated technologies, such as fixed telephony, which are still widely used today.

Technology offers new and creative methods to address vaccination barriers for families, health care providers, and the broader community, due to its prevalence in society and its flexibility [13]. Since 2005, the World Health Organization has urged member states “to develop the infrastructure for [ICT] for health as deemed appropriate to promote equitable, affordable, and universal access to their benefits, and to continue to work with information and telecommunication agencies and other partners in order to reduce costs and make eHealth successful” [14].

Technology use in high-income countries is widespread: the vast majority (92%) of people aged 65 years or older in the United States own a cell phone [15], and this is paralleled by the widespread use of the internet, social media, and email, which creates other promising routes for health communication [13]. In addition to consumer use of technology, healthcare providers are being incentivized to integrate technology into their practice: Coronavirus disease 2019 (COVID-19) accelerated the global adoption of telemedicine [16], and healthcare organizations increasingly offer patients access to their personal electronic health records (EHR) to manage their health [17]. The combination of EHR use, the growing number of immunization registries, and the resulting ability to have vaccination data in electronic form provides an important foundation for delivering ICT-based vaccine interventions [13].

Finally, ICT can offer a cost-effective and accessible way to promote immunizations as an alternative to the traditional means of communication (e.g., letters) or the engagement of health care professionals [18,19].

While previous systematic reviews have assessed the effect of immunization programs [20,21,22,23,24], including technology-based programs [25,26], to the best of our knowledge, no review has specifically focused on ICT use in the older adult population.

The aim of this systematic review is to evaluate recent literature on interventions that apply ICT to improve the uptake of the influenza, pneumococcal, COVID-19, and herpes zoster vaccines among older adults.

## 2. Methods

The review methods were defined in advance, following the Prepared Items for Systematic Reviews and Meta-Analysis (PRISMA) guidelines [27]. Studies were included in the review if they met the following criteria:

(a) Were focused on increasing at least one of the universally recommended vaccinations for older adult populations in high-income settings: the pneumococcal vaccine, the seasonal influenza vaccine, the herpes zoster vaccine, or the COVID-19 vaccine [2].
(b) Investigated the use of an ICT-based intervention to increase vaccination coverage.
(c) Included participants over 60 years old.
(d) Were original studies using a before-and-after, quasi-experimental, or experimental study design (i.e., guidelines, reviews, letters, and editorials were excluded).
(e) Were published from 1 January 2000 to 10 November 2022.
(f) Were written in English.
(g) This systematic review does not include studies involving specific vaccinations delivered exclusively to subgroups of older adults, such as people with specific medical conditions (e.g., the Hemophilus vaccine for splenectomy patients) or international travelers.

### 2.1. Search Strategy

The electronic databases Pubmed and Scopus were used to find original studies. The reference lists of reviews that were found during the search were also hand-searched for relevant studies.

The search strategy was built using a combination of keywords for the 4 main axes of our research question: (1) the selected target population (older adults), (2) the selected interventions (those applying ICT strategies), (3) the selected vaccinations (universally recommended vaccines for older adults), (4) the selected outcome (evaluation of immunization rates). Within each axis, we combined keywords with the “OR” operator and we then linked the search strategies for the two axes with the “AND” operator. The search strings are reported in Appendix A.

### 2.2. Study Selection and Data Extraction

Identified studies were independently reviewed for eligibility by two authors (GG, MT) in a two-step-based process: an initial screen was performed based on title and abstract, then full texts were retrieved for a second screen. At both stages, disagreements between the two reviewers were resolved by consensus or by consulting another author (AB).

A data extraction form based on the research question was developed in Microsoft Excel. Data were collected on the following study characteristics: (1) author name, year, and country of publication; (2) study design; (3) study setting, study period and study population; (4) vaccines considered; (5) the type of ICT applied; (6) the intervention description and aims; (7) outcomes of interest; and (8) author conclusions.

We performed descriptive analysis to report the characteristics of the included studies. To summarize the findings on the effectiveness of each intervention across multiple studies, we displayed the results of individual studies. If unadjusted and adjusted outcomes were available, we recorded the adjusted estimates to reduce confounding factors.

### 2.3. Quality Assessment

The two authors that reviewed the retrieved studies for eligibility (GG, MT) also independently assessed the quality of selected studies using the Cochrane Risk of Bias 2.0 (RoB 2) tool [28] for RCT, the Risk Of Bias In Non-randomized Studies—of Interventions (ROBINS-I) [29] for non-randomized trials, and the National Institutes of Health (NIH) quality assessment tool for before–after (Pre–Post) studies with no control group [30]. Reviewer disagreements were resolved by consensus or by consulting a third author (AB). Appendix B shows the quality assessment assigned to each study.

## 3. Results

### 3.1. Identified Studies

**Figure 1:**
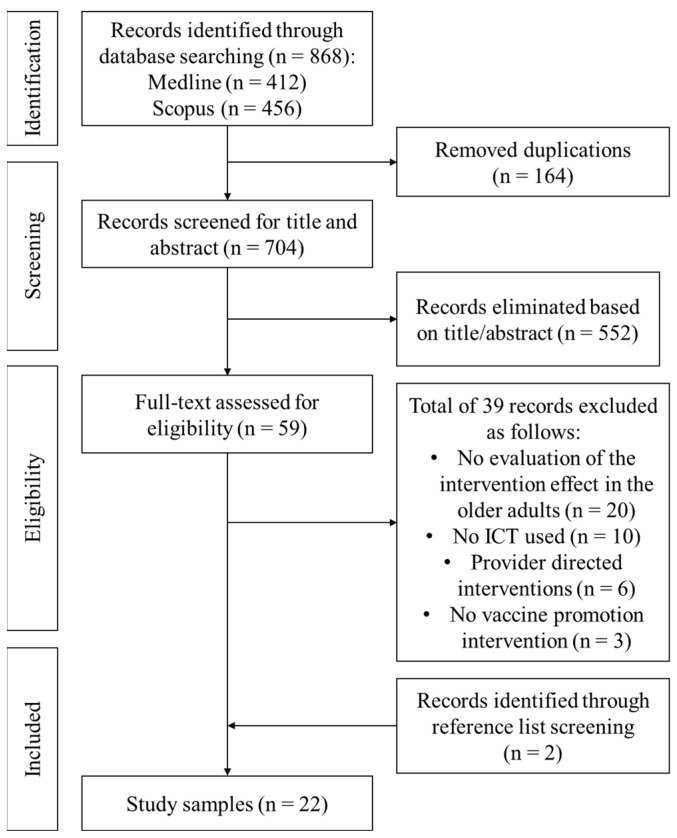
The Preferred Reporting Items for Systematic Reviews and Meta-Analyses (PRISMA) diagram of the article search.

The research strategy returned a total of 868 citations from electronic databases. After removal of duplicates, 704 titles and abstracts were screened and 59 full texts were assessed for eligibility, 20 of which met the exclusion and inclusion criteria. Two additional relevant studies were identified by screening the reference lists of previous reviews, resulting in a total of 22 articles included in this systematic review (Figure 1).

### 3.2. Characteristics of the Included Studies

Major characteristics of the studies identified and included in our review are reported in Table 1. Table 2 shows the results of these retrieved studies.

The majority of the studies (n = 15) were conducted in the United States [19,32,35,36,37,38,39,41,43,45,46,47,48,49,50], three around Europe (Spain [42], the UK [34], France/Monaco [44]), two in the Middle East (Lebanon [33] and Turkey [31]), one in Canada [40], and one in Australia [18].

In terms of the study designs, most interventions (n = 15) were Randomized Controlled Trials (RCT) [18,19,34,35,37,38,39,41,43,44,45,46,48,49], while the others either had a quasi-experimental design [31,36,42] or were before-and-after trials [32,40,47,50].

Almost all interventions involved the general population with the exception of four studies that focused on individuals suffering from specific clinical conditions: asthma or chronic obstructive pulmonary disease [37], patients diagnosed with a selected rare disease [42], patients in a home telehealth program with selected characteristics in terms of clinical issues, dependencies in activities of daily living and social support network [50] or patients with hypertension [39].

Of the 22 included studies, 11 promoted the influenza vaccine [18,34,35,36,39,40,42,43,45,46,50], four promoted both the influenza and the pneumococcus vaccines [37,38,44,48], three the pneumococcus vaccine [31,33,41], one the COVID-19 vaccine [32], two the herpes zoster vaccine [19,47] and one both the pneumococcal and the herpes zoster vaccines [49].

All but one of the studies reported results on vaccine coverage, while only one study [40] exclusively assessed the willingness of people to get immunized. One other study [41] evaluated both outcomes.

Some of the included ICT-based interventions also incorporated the patients’ personal catchment conducted with a traditional method of communication (i.e., postcards, letters, or informational sheets) as follows: (a) in three studies [34,36,44], the entire population (controls and interventions) received a personal catchment with traditional means of communication as well as an ICT-based contact, which was conducted only in the intervention group; (b) in seven studies [30,31,32,34,39,41,42], the personal catchment was conducted only in the intervention group using both ICT strategies and traditional means of communication (with no personal contact in the control group); (c) four studies [19,31,37,39] compared the effects of an ICT-based intervention with an intervention using exclusively traditional means of communication.

A health education component (i.e., a message about the importance and safety of the investigated vaccine) was included in 11 out of the 22 studies [19,31,32,33,38,39,40,41,44,45,50], while the others used an ICT tool merely as a vaccination reminder [18,31,34,36,37,42,43,46,47,48,49].

The following ICT tools were used in the studies investigated: 10 interventions were conducted by telephone calls [31,32,34,35,36,37,38,39,40,41], four used text messages [18,42,43,44], three sent messages via electronic health systems [19,45,46], three used autodial calls [47,48,49] and remote patient monitoring in a home telehealth program [50], and one study included a mix of tools (telephone calls, text messages, and emails) in different subgroups [33].

### 3.3. Telephone Calls

Among the 10 intervention studies conducted by means of telephone calls, six were RCTs [32,35,36,38,41,46], two were non-RCTs [34,49] and two were before-and-after studies [31,50]. Among the controlled studies, six out of eight showed a good level of efficacy, with vaccination rates in the intervention group exceeding those in the control group by 6.3 to 42 percentage points [32,36,38,41,46,49]. With regard to the two before-and-after studies, one of them was effective in increasing vaccination rates by 40 percentage points [50], while the other had a very small effect (three new patients vaccinated out of 59) [31].

Telephone calls were applied in two different kinds of interventions: (a) dialogue-based interventions with a relevant educational component, and (b) patient reminders or invitations to schedule an immunization appointment.

(a) Dialogue-based interventions with an educational component.

Among the studies conducted with telephone calls, six out of 11 offered a dialogue-based intervention with a relevant health educational component that provided information about the importance and safety of the vaccine and assessed participants’ main concerns [31,32,38,39,40,41]. Three of these were RCT [38,39,41] and all saw higher immunization rates in the intervention groups compared to control groups.

In particular, Krieger et al. [38] aimed at increasing pneumococcal and influenza immunization rates among urban seniors using peer-to-peer outreach. The intervention group received educational brochures in the mail and was contacted by volunteers to encourage immunization and address specific barriers, while the control group was exposed to the usual senior/community immunization promotion activities. The intervention was effective, particularly in increasing pneumococcal immunization rates (intervention group: 52.0%, 95% CI [46.6–57.4]; control group: 30.9%, 95% CI [26.6–35.2]; rate ratio = 1.68, 95% CI [1.40–2.03]). The intervention was also effective for influenza vaccination among those that were not immunized in the previous year (intervention group: 50.0%, 95% CI [40.0–60.0]; control group: 23.0%, 95% CI [15.2–33.3]; rate ratio = 2.17, 95% CI [1.42–3.31]), while the rate ratio was lower among those that were immunized in the prior year but remained significant (rate ratio = 1.04, 95% CI [1.01–1.07]).

Winston et al. [41] evaluated the effectiveness of adding a telephone intervention conducted by a nurse (just in the intervention group) to a pre-existing reminder letter (received by both interventions and controls) with the goal of increasing pneumococcal vaccination in a target population. The added contact by phone was successful at increasing vaccination rates, with 17% (201/1198) of the intervention patients being vaccinated versus 8% (100/1197) of the controls, at 6-month follow-up.

Minor et al. [39] evaluated the effectiveness of both mail (first intervention group) and telephone (second intervention group) communication strategies, comparing them to a control group (which received standard clinical approaches). This was conducted with patients of a hypertension clinic and was aimed at improving existing influenza vaccination rates among those not seeking early seasonal vaccination. Both the intervention groups were more effective than the control, with the phone group resulting in higher vaccination rates (females: control group 50.0%, mail 54.4%, phone: 58.9%; males: control group 58.3%, mail 58.3%, phone 87.5%).

Three telephone-based interventions with an education component had a pre–post intervention design [32,33,40]. Desir et al. [32] reported a tailored, dialogue-based intervention aimed at increasing COVID-19 immunizations in older adults in an outpatient geriatric clinic who were identified as possibly not having been vaccinated in the summer and fall of 2021 (information from electronic medical records). The interventions provided relevant information and/or logistical assistance. Of 59 non-vaccinated older adults, the patients who had expressed interest in vaccination (n = 20) were re-contacted approximately 1 month later to ask about their updated vaccination status: as a result, four (6.8%) received the COVID-19 vaccination at the end of the intervention.

Strain et al. [40] reported a telephone script-based intervention created to guide a brief (1–3 min) discussion on influenza vaccination between pharmacy staff and unvaccinated individuals. This was conducted predominantly at the “tail end” of the vaccination season, in order to target those who did not respond to initial invitations from their primary care physicians. The main outcome of the study was for contacted individuals to have a vaccine administration appointment arranged by the end of the phone consultation (i.e., showing willingness to get immunized). Among 474 individuals that did not have documented influenza vaccination, 316 (67%) agreed to receive the vaccine by the end of the consultation [40].

Ghadieh et al. [33] looked at pneumococcal immunizations, analyzing the effect of three different types of patient reminder system (short phone calls, text messages, and e-mails), with the addition of a brief education intervention about the seriousness of pneumococcal disease in half of the subgroups (for a total of six intervention subgroups). Each subgroup received three identical reminders given at four-week intervals, in which patients were asked to call the clinic themselves and schedule an appointment to receive the vaccine. The rates of vaccination in patients older than 65 years increased from 17.2% to 20.4% before and after the intervention, but the study did not provide any specific data comparing the intervention subgroups to a historical control. The authors reported that telephone calls have a greater impact than email or SMS in the overall sample (which also included younger adults), and that all intervention groups have better results than the control group (i.e., no intervention). There was also no statistical significance when stratifying the results according to age.

Lastly, Biyik et al. [31] conducted a quasi-experimental study assessing the impact of doctor recommendations through face-to-face interviews (for patients who attended a family health center, n = 103 patients) or by phone (for patients who did not attend the clinic, n = 97 patients) on pneumococcal vaccination rates. They reported that 59.8% of patients contacted by telephone received the vaccine versus 81.6% of those that were interviewed face-to-face.

(b) Patient reminders/recalling and invitations to schedule an appointment to get immunized.

Four studies (three RCT [34,35,37] and one non-RCT [36]) contacted individuals by telephone, inviting them to schedule an immunization appointment or reminding them about the opportunity to receive a certain vaccine. Three studies focused on influenza immunization [34,35,36] and one on both the influenza and pneumococcal vaccines [37]. Two of these studies reported higher immunization rates in the intervention group than in the control group [34,35].

Humiston et al. [35] provided a multicomponent intervention based on: (1) GP reminders, (2) patient recalling (mailed influenza immunization reminders in the form of a letter or card), and (3) patient outreach (phoning patients who had no routine appointment scheduled during the 3-month flu vaccine period and asking them to make an appointment). Patients in the intervention group were more than six times more likely to be vaccinated than patients in the control group (odds ratio = 6.25, 95% CI [5.41–7.22], *p* = 0.0001).

Hull et al. [34] reported an intervention directed at low-risk patients aged 65 to 74 years who had not previously been in a recall system for influenza immunization. The intervention was based on a telephone call from the receptionist, offering an appointment to receive the vaccine. With this, immunization rates were boosted by about 6.3% (*p* = 0.026).

On the other hand, some studies found no effect of these interventions. For instance, Kellerman et al. [36] conducted an intervention on non-responders from a family practice center that had already received a postcard urging influenza immunization in the previous month. The intervention group was contacted by phone but no significant increase in immunization rates was found compared to the controls.

Similarly, Klassing et al. [37] looked at influenza and pneumococcus immunization rates, comparing the effect of pharmacy-initiated interventions directed at asthma or COPD patients. Patients were randomized into one of the three study arms: phone calls, mailed letters, and controls. No significant difference was found between the interventions and the control group for the over-65 population.

### 3.4. Text Messages

The four interventions that applied text messages were three RCTs [18,43,44] and one non-RCT [42]. Three of these exclusively used text messages to invite/remind patients to get immunized against influenza, without any other traditional means of communication and without offering any kind of health education program [18,42,43]. Of these, one RCT increased vaccination rates by five percentage points compared to controls [18], two were only partially effective because they increased vaccination coverage only in specific population subgroups or only for certain vaccinations [45,47], and one had no significant effect [37].

Two of these studies were conducted in primary care settings [18,43]. Regan et al. [18] found that the use of an SMS reminder, sent 6 weeks after the influenza season began, increased vaccination rates by 5% for patients 65 years of age or older (20.5% vaccinated, n = 376 in the intervention group vs. 15.8% vaccinated, n = 281 in the control group, RR = 1.26, 95% CI [1.10–1.45], *p* < 0.05). Patel et al. [43] also evaluated the effect of text messaging: 19 different protocols (nudges) were developed by behavioral scientists to boost influenza vaccination rates during routine primary care visits; nudges varied in their message contents and/or timing (up to two sets of messages were sent from the patient’s healthcare provider in the 3 days preceding the patient’s appointment). Although some nudge protocols were effective among the overall population of the study, and the subgroups analysis by patient age showed no significant differences among the age subgroups, none of the nudge protocols was significantly effective among the patients aged 65 years of age or older. The third study was conducted by Esteban-Vasallo et al. [42], assessing the effect of text message reminders on influenza vaccination uptake in patients with selected rare diseases and delayed vaccination status. The authors found that, for older adults, the reminder was associated with a significantly higher vaccination probability in patients that had at least one concurrent chronic condition in addition to the rare disease (IRR = 1.23, CI 95% [1.08–1.40]). When further analyzing by sex, the authors report that the intervention was effective for men with at least one concurrent chronic condition in addition to the rare disease (IRR: 1.58, CI 95% [1.25–2.00]) and for women without concurrent chronic conditions (IRR: 1.40, CI 95% [1.05–1.89]). Lastly, Tubiana et al. [44] reported a multifaceted intervention aimed at increasing pneumococcal and influenza vaccination rates in patients aged 65 years and older that had gone to an Emergency Department. Patients in the experimental group received a brief structured interview with the physician about issues associated with pneumococcal and influenza infections and the importance of both vaccinations. They were also given an information sheet with an explanation about the risks and benefits of vaccination and a letter dedicated to the general practitioner stating that the patient was at risk for pneumococcal infection and could benefit from pneumococcal vaccination. In addition, three text messages were sent to the patients every 2 weeks to remind them to talk about pneumococcal risks with their general practitioners. Patients in the control group had a similar intervention, except that they did not receive the text message reminders and they had a non-structured interview about pneumococcal risks and vaccination. The primary outcome was self-reported pneumococcal vaccination within 6 months, with 6-month self-reported influenza vaccination being evaluated as a secondary outcome. In the intention-to-treat analysis, the intervention did not alter the pneumococcal vaccination rate (6.4% vs. 4.6%, absolute difference: 1.8, 95% CI [−0.9–4.4], *p* = 0.19), but it did improve the influenza vaccination rate (52.1% vs. 40.0%, absolute difference: 12.1, 95% CI [2.4–21.8], *p* = 0.01).

### 3.5. Automated Phone Calls

Three interventions used automated phone calls: two of them were RCTs [48,49], and one had a before-and-after design [47]. One of the RCTs increased vaccination rates in the intervention group by 1.5 percentage points compared to the control group [33], while the other had no significant effect [43]; the before-and-after study showed a higher number of vaccinations during the intervention period compared to the control period (25 vs. 16) [42].

Hurley et al., conducted a RCT using auto-dial calls as centralized patient reminders/recalls to increase seasonal influenza, pneumococcal, and Tdap vaccines (not considered in this review) [48]. Over 3–4 months, participants received up to two auto-dial phone calls followed by a postcard indicating that they may need one or more vaccines (without specifying what vaccines were needed), encouraging them to schedule an appointment or to go to a pharmacy and get immunized. The intervention was effective in increasing influenza vaccination in older adults, with 32.0% (n = 847) receiving a vaccine in the intervention group versus 28.6% (n = 760) in the regular-care group (*p* < 0.01). No significant difference was found for the pneumococcus vaccine.

The other RCT, conducted by Stolpe et al. [49], found no significant increase in pneumococcal and herpes zoster vaccination rates among community pharmacy patients who received an automated telephone call offering the vaccines compared with control group patients who received a scheduled outbound communication without the added vaccination prompt.

Also aiming to promote herpes zoster vaccine among patients of a community pharmacy, Bedwick et al. found, in a before-and-after trial [47], that a total of 25 herpes zoster vaccines were administrated during the intervention period, compared with 16 administrated during the same period in the previous year.

### 3.6. Personal Electronic Health Records

Three of the RCTs were conducted using EHR [19,45,46]. One of them increased vaccination rates in the intervention group by eight percentage points compared to the control group [19], while the others had no effect or showed only small significant improvements in a specific sub-analysis [39,40]. Otsuka et al. [19] included patients from a general internal medicine clinic aged 60 years and older, assessing the effect of giving an informational packet (regarding shingles and the herpes zoster vaccine) through EHR or through the national postal service, comparing their standard of care. At their 6-month follow-up, both strategies were effective in increasing vaccination rates compared to the controls (relative risk for the EHR group: 2.7, 95% CI [1.6–4.5], *p* = 0.0001; relative risk in the non-EHR group: 2.9, 95% CI [1.6–5.5], *p* = 0.0007) and the outcome of the logistic regression interaction likelihood ratio test revealed that the effects of the two interventions were not significantly different (*p* = 0.99).

The other two interventions using EHR were conducted by Szilagyi et al. in 2020 [45] and 2022 [46]. Both studies aimed at increasing influenza vaccination rates using messages sent via the healthcare system’s EHR, where the first study provided relevant information about vaccine safety and effectiveness [45], and the second used messages tailored to patient characteristics (i.e., age and any chronic diseases) and incorporated behavioral science strategies [46]. The primary outcome of both studies was to register influenza vaccination in the EHR after the intervention, while the secondary outcome also included self-reported vaccination by the patients in response to a portal reminder (this option was only available for the intervention group). Both studies found no significant effect on the primary outcome, but when self-reported vaccination was taken into account, the first study found a significant improvement in vaccination rates with a dose–response increase depending on the number of reminders sent (vaccination rates: 53.6% in the control group, 54.6% in the one-reminder group, 55.1% in the two-reminder group, and 56.7% in the three-reminder group; *p* < 0.001) [45]. In the second study, no significant difference was noted in influenza vaccination rates, but adjusted multivariate analyses showed a small effect for the “loss frame” behavioral science strategy (in which the message describes what a person has to lose by taking a particular action; RR = 1.03, 95% CI [1.01–1.05], *p* < 0.01) [46].

### 3.7. Remote Patient Monitoring in a Home Telehealth Program

One study with a before-and-after design aimed to improve seasonal influenza vaccine rates among patients with specific clinical issues, dependencies in activities of daily living, or a poor social support network who were part of a home telehealth program [43]. Automated two-way messaging offering the influenza vaccination was transmitted using remote patient monitoring, and tailored education was provided during a telephone visit for patients that had no vaccination plans. As a result, 81.7% of patients aged 66 years and older received the vaccine during the intervention period, but the intervention was insignificant in increasing vaccination rates compared with the previous flu season (control period).

## 4. Discussion

This systematic review summarizes the data from ICT-based interventions aimed at increasing immunization rates for recommended vaccines in older adults. The 22 retrieved studies are heterogenous in terms of the included populations (i.e., some focus on the general population while others focus on individuals suffering from a specific disease or vaccine-hesitant individuals) and are also heterogenous in terms of the intervention strategies (i.e., simple reminders/recalls, educational interventions, traditional means of communication in addition to the ICT tool, or multicomponent interventions).

Overall, half of the included studies (n = 12) found some level of effectiveness of ICT strategies in increasing vaccination rates among older adults [18,19,31,33,34,35,38,39,40,41,47,48]. In particular, higher effects were found in one study that used patient recall and provider prompts, leading the intervention group to be more than six times as likely to receive the influenza vaccine [35]. The highest effect of this intervention is probably due to its multi-component approach (mailed reminders for flu immunization plus up to two phone calls to patients who had not scheduled a routine appointment) and the high level of GP engagement through the reminders (patients’ charts were marked with a reminder to the doctor that the patient had not been vaccinated).

Another intervention using telephone contacts plus educational brochures mailed to the participants greatly increased both influenza (50.0% intervention vs. 23.0% control) and pneumococcal (52.0% intervention vs. 30.9% control) immunization rates [38]. It is worth noting that interventions using leaner ICT strategies (such as electronic messages or autodial calls) that cost less in terms of time and human resources found good levels of effectiveness. For instance, Regan et al. [18] and Otsuka et al. [19] found a respective 5% and 8% improvement in vaccination rates in the intervention groups compared to controls.

Other interventions (n = 5) were only partially effective, finding an increase in immunization rates only for specific vaccines or for population subgroups [32,42,44,45,46]. One example is a multicomponent intervention with a consistent educational component that revealed that text message reminders were effective in increasing influenza but not pneumococcal vaccination rates [44]. It should be noted that this study enrolled patients visiting an emergency department after their acute medical condition was resolved; thus, they could have been reluctant and not so interested in the prevention program. Furthermore, awareness of the need to recommend pneumococcal vaccination is probably lower among both physicians and patients than for the influenza vaccine [51]. Other authors have also reported that external factors may hamper pneumococcal vaccination, such as problems in pneumococcal vaccine supply [44]. Two other studies that were partially effective were conducted by the same authors, in a similar population, using EHRs over two consecutive different years (2018–2019 and 2019–2020) [45,46]. One possible reason for the lack of impact in these studies is that not all the patients opened the portal messages, so perhaps strategies that encourage patients to read these reminders might be helpful. Another included study was successful in implementing vaccination rates by using EHR messages [19]; however, further studies are needed to clarify the role of this technology in vaccination promotion. Similarly, another partially effective study [42] aimed at increasing influenza immunization using text messages (in this case, patients had certain rare diseases as well as delayed vaccination) and found that receiving the reminder was associated with a significantly higher probability of vaccination in patients with at least one concurrent chronic condition in addition to their rare disease. This higher vaccination uptake could be related to a higher perception of risk, as well as more frequent contact with health facilities, thus increasing the opportunities for vaccination. Although the intervention was only modestly effective, the authors suggest that it might be a good additional strategy to improve vaccine uptake, since it is simple, feasible, affordable, and easily scalable, particularly when immunization and target population data are available in digital registries.

Lastly, five studies found no significant effect of the intervention [36,37,43,49,50]; these interventions varied in terms of the technology used, which included text messaging, phone calls and automated phone calls. One non-effective telephone-based study focused on non-responder patients of a family practice that had already received a mailed invite to get immunized against influenza the month before [36]. In this case, the lack of an effect is likely to be influenced by the previous mail-based intervention—this being effective, it would have left a population of individuals that were more hesitant towards the vaccine, probably less aware of influenza risks, and generally less motivated to be immunized, meaning that they were not influenced by the intervention. Also, an educational intervention conducted on home telehealth patients revealed no significant improvement on influenza vaccination rates compared with the previous flu season: however, it is important to note that vaccination rates among this particularly frail population were already quite high, with more than 80% of older adults vaccinated [50].

Another non-effective study was a telephone-based pharmacist-initiated three-arm trial focusing on individuals suffering from asthma or chronic obstructive pulmonary disease [37]. The sub-analysis conducted for patients over 65 years of age found no significant difference in pneumococcal and influenza vaccine rates between the two intervention arms (a phone call and a mailed letter) and the control group. This study saw a high vaccination rate within the control group that may have come from a high baseline level of vaccination within the pharmacy populations, largely due to both the socioeconomic status of the study participants, and the high number of vaccination advertisements during the study time period [37]. In each of the three involved pharmacies, printed advertisements promoting in-store immunizations were given with each prescription for the duration of the study period and a new CDC recommendation for the pneumococcal vaccine was released during the same period, which was highly publicized in print and on TV. Furthermore, during the course of the phone call intervention, many patients expressed the desire to discuss the vaccine recommendations at their next physician appointment (after the conclusion of the study period), suggesting that patients in the letter group may have had this same idea [37]. Another intervention using automated calls to improve pneumococcal and herpes zoster vaccines revealed low call completion rates, with 28.2%, 6.2% and 1.0% of call attempts completed for the first, second and third attempts, respectively, which is probably the main reason for the inefficacy of the intervention itself [49]. Finally, a study prompting influenza immunization using 19 different text messaging (nudges) protocols found that none of them was effective in increasing vaccination rates among older adults, although some were effective among the overall population, and subgroups analysis by patients’ age showed no significant difference between the older and the younger groups [43]. In addition, because a previously published interim analysis of the same study, reporting only data of patients enrolled in the fall of 2020, showed a higher overall effect among the overall population and a significant effect of a nudge protocol among older adults [52], the authors hypothesized that the release of the COVID-19 vaccine that began in January 2021 may have influenced influenza vaccination rates in the trial.

The acceptance and rejection of vaccines is highly context-dependent and is influenced by ‘local vaccine cultures’, social, cultural, historical and political factors [53]. Most of the studies included in this review were conducted in countries that offer free or insured vaccines for the elderly population, and the lack of an economic barrier probably improves vaccination uptake. This is supported by the results of one of the included studies [47], in which both pneumococcal and influenza vaccination were promoted, but the intervention was only effective for the influenza vaccine, which was the only one offered free of charge. On the other hand, it is worth noting that the only study in which the vaccine was paid for out of pocket by the participants also proved effective [48]; this could be explained by the fact that it was conducted in a middle-income country in which vaccine acceptance is generally high, mainly due to an increased interest in personal protection against infectious diseases and trust in health professionals as sources of guidance [54]. In addition, the setting of the vaccination service and/or vaccine promotion activity could influence vaccination uptake; for example, this review included five studies in which the vaccine promotion intervention was based on telephone calls [35,38,50] or automated calls [42,43] and conducted by pharmacists; three of these were found to be effective in increasing vaccination rates, a slightly higher rate of effectiveness than that found in interventions conducted in primary care centers (six out of 11 effective studies). Despite the low number of studies and the need for further research, community pharmacies could contribute to achieving vaccination goals by increasing vaccination rates due to their multiple locations in metropolitan and urban areas, convenience, extended opening hours and reduced costs for vaccine administration [55].

Despite the use of ICT communication methods, which are particularly novel when considering non-telephone-based studies, the majority of the interventions seemed to be effective in older adults, similarly to what was seen in another study that looked at different ages [56]. This could be partly due to their acceptance of commonly used technologies [57], and also because patients with greater difficulties in the use of ICT can provide a close relative as their contact through the healthcare system.

Overall, ICT tools can be an additional strategy that could be used in health promotion campaigns to improve vaccine uptake in older adults, delivering information directly to the individuals at a relatively low cost.

## 5. Limitations

This systematic review has some limitations. Firstly, the included studies are heterogeneous in terms of their study setting, study populations, and type of intervention applied (e.g., the presence or absence of an educational component). This limits the potential of quantitatively pooling estimates and prevents meta-analysis. Secondly, many interventions are multicomponent, also including communicative strategies that involved traditional means of communication on top of the ICT, making it difficult to estimate the true effect of the technological tool alone. Finally, in this systematic review, the vast majority of RCTs were assessed with a low risk of bias (overall low risk of bias for 12 out of 15 studies, some concern for one and a high risk of bias for another). However, among the interventions with a study design other than RCTs (three non-RCTs and the four studies with before-and-after designs), the quality was lower on average, with four out of seven studies rated as being of low or moderate quality.

## 6. Conclusions

The ICT field has grown exponentially in recent years, becoming widely accessible to the general population. This review showed that ICT-based interventions could help to increase immunization coverage among older adults, with the majority of the included studies finding some level of effectiveness. However, further research is needed to assess how ICT can be optimally used to convey truly effective messages that improve public and individual health.

## Figures and Tables

**Table 1:** Overview of included studies: characteristics, materials and methods.

Main Author, Year, Setting	Study Design	Aim	Sample Size and Characteristics of the Sample (Age Distribution). If Not Otherwise Specified, the Sample Is from the General Population	Vaccine(s)	Traditional Means of Communication	Educational Component
PHONE CALLS						
Biyik et al., 2020, Turkey (Ankara) [31]	Non-RCT	To observe the impact of doctor recommendations on the phone compared to face-to-face interviews.	Two hundred patients aged 65 years and older registered at an education family health center. Average age was 72.26 years.	Pneumococcal	No	Yes
Desir et al., 2022, United States (Miami) [32]	Before-and-after trial	To address COVID-19 vaccination barriers with a tailored, dialogue-based intervention also providing logistical assistance.	Fifty-nine unvaccinated patients of a geriatric primary care clinic. Vaccine-hesitant population, Average age: 77.7 ± 7.1 years.	COVID-19	No	Yes
Ghadieh et al., 2015, Lebanon (Beirut) [33]	Randomized allocation for the intervention arm and before-and-after analysis of the results	To evaluate and compare the effect of different types of patient reminder (text messages, e-mails, short phone calls) to improve pneumococcal immunization.	Unvaccinated active patients (who had at least one visit to the clinic in the last 12 months) of a family medicine center. Patients excluded from the study had no e-mail or phone number recorded; family members sharing the same contact information were categorized into the control group.	Pneumococcal	No	Yes, for 3 out of the 6 study arms
Hull et al., 2002, UK (East London and Essex) [34]	RCT	To determine whether telephone appointments offered by general practice receptionists increased influenza immunization in population aged 65–74 years old.	A total of 1820 low-risk patients from 3 general practices. In each practice, the study population was condensed to a list of households, which were randomized into either the control or intervention group. Mean age: intervention group 69.2 years, control group 69.3 years.	Influenza	National and community advertising and mail campaigns	No
Humiston et al., 2011, United States (Rochester) [35]	RCT	To evaluate the effect of a multicomponent practice-based intervention on (1) influenza immunization rates and (2) disparities in vaccination rates by race/ethnicity and insurance status.	A total of 3752 active patients aged >65 years in 6 primary care practices. Control group n = 2004; intervention group n = 1748. Age distribution: 65–69 years: control n = 663 (33%), intervention n = 589 (34%); 70 years or more: control n = 1341 (67%), intervention n = 1159 (66%).	Influenza	Postcard/letters and provider reminder (alert in the patients’ chart at the GP)	No
Kellerman et al., 2000, United States (Kansas) [36]	Non-RCT	To determine if postcard and telephone reminders increased the rate of influenza immunization.	A total of 475 non-institutionalized patients of a community family practice center aged 65 years or older received a postcard urging influenza immunization. Those not responding within 1 month were systematically allocated either to a group receiving further telephone contact (n = 154) or to a control group (n = 216). Vaccine-hesitant population.	Influenza	Postcard mailed to the entire population	No
Klassing et al., 2017, United States (Kansas City) [37]	RCT	To investigate if pharmacy-initiated interventions (outbound phone calls or mailed letters) improved influenza and/or pneumococcal vaccination rates among adult patients with asthma or COPD, compared to controls.	A total of 831 adult patients from three pharmacies diagnosed with asthma and/or COPD were randomized into 3 study arms. [Note: Only the sub-analysis for the over-65 population was considered in this review].	Influenza, pneumococcal	No	No
Krieger et al., 2000, United States (Washington) [38]	RCT	To increase pneumococcal and influenza immunization rates among an urban senior population using peer-to-peer outreach.	Individuals aged 65 years or older were recruited from a senior center and a marketing database. Control group: n = 624, mean age 75.1 years. Intervention group: n = 622, mean age 75.6 years.	Influenza, pneumococcal	Educational brochure	Yes
Minor et al., 2010, United States [39]	RCT	To assess the effectiveness of mail or telephone reminder strategies on improving existing influenza vaccination rates among those not seeking early seasonal vaccination.	Patients at a hypertension clinic without record of recent influenza vaccination. Two intervention groups (a mail reminder or a phone reminder) and a control group (standard clinical practice).	Influenza	No	Yes
Strain et al., 2021, Canada (Alberta) [40]	Before-and-after trial	To increase influenza immunization in individuals 65 years of age and older by facilitating conversations about influenza vaccination with consumer-facing pharmacy staff.	A total of 474 unvaccinated individuals aged 65 years or over identified from the dispensing software of 28 pharmacies. Vaccine-hesitant population.	Influenza	No	Yes
Winston et al., 2007, United States (Atlanta) [41]	RCT	To determine the effectiveness of a telephone reminder to increase pneumococcal vaccination in a population that had received mailed reminders.	Unvaccinated adult patients at 5 general medicine clinics with chronic medical conditions and 65 years and older without chronic medical conditions. For the senior group (n = 2395): intervention: n = 1198, age 72.0 ± 0.2 years; control: n = 1197, age 71.4 ± 0.2 years Vaccine-hesitant population.	Pneumococcal	Mailed letter encouraging patients to schedule the pneumococcal vaccination	Yes
TEXT MESSAGES						
Esteban-Vasallo et al., 2019, Spain (Madrid) [42]	Non-RCT	To assess the effect of text message reminders on influenza vaccination uptake of patients with selected rare diseases and delayed vaccination.	Patients diagnosed with a rare disease with indication for influenza vaccination (patients aged 65 years and over n = 45,074). Control group: the individuals with a report of a “pending” message, “unknown number”, or without an available mobile telephone number.	Influenza	No	No
Patel et al., 2022, United States (Northeastern) [43]	RCT	To identify whether and how text messaging interventions (19 different nudges) could be used to boost influenza vaccination rates at routine primary care visits.	All unvaccinated patients with new or routine primary care appointments with a registered mobile telephone number.	Influenza	No	No
Regan et al., 2017, Australia [18]	RCT	To investigate the impact of using text messages to encourage seasonal influenza vaccination among patients at family practice clinics.	Unvaccinated adult patients eligible for influenza vaccination with a registered mobile telephone number. For individuals aged 65 years and older: intervention group n = 1781, control group n = 1832.	Influenza	No	No
Tubiana et al., 2021, France and Monaco [44]	RCT	To determine whether a multifaceted intervention directed to patients attending the emergency department (ED) could increase pneumococcal and influenza vaccination rates at 6-month follow-up.	A total of 1475 patients aged 65 years and older attending an ED. Exclusion criteria: refusal to participate, inability to receive text messages, dementia, altered mental status, language restriction, previous pneumococcal vaccination, or contraindication to pneumococcal vaccination. Intervention arm: n = 780; control arm: n = 695. Median age: 74 years; interquartile range = 69–82.	Influenza, pneumococcal	A brief structured interview with the ED physician, an information sheet for the patient, a letter to the General Practitioner (GP)	Yes
ELECTRONIC MESSAGES SENT VIA PERSONAL HEALTH RECORD						
Szilagyi et al., 2020, United States (Los Angeles) [45]	RCT	To evaluate the effect of patient reminders on influenza vaccination rates. Reminders were sent via an electronic health record patient portal.	A total of 164,205 patients in 52 primary care practices who had used the patient portal within 12 months. Exclusion criteria: individuals who were not active portal users and family members of index patients. Of the individuals, 18.7% were 65 years or older.	Influenza	No	Yes
Szilagyi et al., 2021, United States (Los Angeles) [46]	RCT	To evaluate the impact of a health system’s portal reminders on influenza vaccination rates among adults. Reminders were (1) tailored to patient characteristics, and (2) incorporated behavioral science strategies.	Patients in 53 primary care practices who had used the patient portal within 12 months. Exclusion criteria: individuals who were not active portal users and family members of index patients. Age distribution for the ≥65 year group (n = 29,795): mean = 73.6 years (SD = 7.2), median = 71.8 years (Q1 = 68.0, Q3 = 77.2), min = 65.0 years, max = 106.7 years.	Influenza	No	No
Otsuka et al., 2013, United States (Ohio State University) [19]	RCT	(1) To determine whether the herpes zoster vaccination rate could be increased by communicating with the patient outside of an office-based face-to-face visit. (2) To compare intervention effects on patients that were sent communications via the US postal service with those sent communications via their personal health record.	Unvaccinated patients aged 60 years and older were stratified on the basis of activated personal health record status. Among each group, participants were randomized into the intervention group or the control group. Personal health record users: Intervention group: n = 250, mean age 69.8 (SD 8.3) years. Control group: n = 424, mean age 68.6 (SD 7.9) years. Non-Personal health record users: Intervention group: n = 250, mean age 74.4 (SD 10.0) years. Control group: n = 1665, mean age 74.0 (SD 9.8) years.	Herpes zoster	No	Yes
AUTOMATED PHONE CALLS						
Bedwick et al., 2017, United States (Morgantown) [47]	Before-and-after trial	To assess the impact of an automated telephone call by a pharmacy owner on the number of herpes zoster vaccinations given and to compare the number of herpes zoster vaccinations in the 3 months during the previous year with the 3 months of the intervention.	All patients of a community pharmacy (approximately 600) who were 60 years of age and older had a prescription filled at the pharmacy during the past year and had a telephone number on file with a local area code.	Herpes zoster	No	No
Hurley et al., 2018, United States (Denver) [48]	RCT	To assess the effectiveness and implementation costs of centralized vaccine reminders/recalling for adult seasonal influenza, pneumococcal, and Tdap [not considered in this review] vaccines.	Patients of an integrated healthcare system; 5332 individuals aged >65 years old were split evenly between intervention (n = 2665) and control (n = 2667) arms. Age distribution of the over-65 group: 65–79 years: intervention 86%, control 85%. Over 80 years: intervention 15%, control 15%.	Influenza, pneumococcal	Mailed postcards (only to the intervention group)	No
Stolpe et al., 2019, United States (Northeastern) [49]	RCT	To determine the effect on vaccination rates of an automated telephonic intervention for adults in need of either pneumococcal vaccination or herpes zoster vaccination, or both.	A total of 22,301 unvaccinated patients who were scheduled to receive an automated telephone call from their community pharmacies. Eligibility criteria: (a) individuals aged either at least 65 years or between 19 and 64 years with potentially high-risk conditions for pneumococcus infection; or (b) individuals aged at least 60 years with no herpes zoster vaccination. Average age = 63 years. [Note: only the sub-analysis for the over-60 population was considered in this review].	Pneumococcal Herpes zoster	No	No
REMOTE PATIENT MONITORING IN A HOME TELEHEALTH PROGRAM						
Rand et al., 2022, United States (San Francisco) [50]	Before-and-after trial	To improve seasonal flu vaccine rates using novel home telehealth clinical and technology interventions	Patients enrolled in a Home Telehealth program (n = 513) from 17 September 2019, to 15 March 2021. Participants met specific criteria regarding clinical issues, dependencies in activities of daily living, or poor social support network; 97% percent of the cohort was male, and over one-half aged from 70 to 79 years.	Influenza	No	Yes

**Table 2:** Overview of included studies: results.

Main Author, Year, Setting	Intervention	Results	Conclusions
PHONE CALLS			
Biyik et al., 2020, Turkey (Ankara) [31]	Patients were informed about the importance of pneumococcal vaccines and were asked about their attitude towards being vaccinated, which was done by phone call (n = 97) or by face-to-face interview (n = 103). The patients who agreed to be vaccinated were prescribed the polysaccharide pneumococcal vaccine at the family health center.	Before the intervention, 2.5% of the elderly patients were previously vaccinated. Fifty-eight of 97 patients (59.8%) who completed the questionnaire during a phone call and 84 of 103 patients (81.6%) who completed the questionnaire during a face-to-face interview received the pneumococcal vaccine.	Immunization rates increased when doctors provided consultation to participants about adult immunization.
Desir et al., 2022, United States (Miami) [32]	Semi-structured conversations to assess patient vaccination status at that time. For patients who confirmed that they were unvaccinated, a tailored intervention intended to address his/her specific barriers to vaccination was attempted, using predetermined options for questions regarding vaccination barriers. Approximately 1 month later, patients who had expressed an interest in vaccination were recontacted (n = 20) to ask about their updated vaccination status and to explore whether there were new or continued barriers to vaccination.	Only 3 patients raised physical and/or cognitive concerns that were, in association with limited social support, limiting their access to vaccination. Logistical support was offered and 30 days later, one of the three patients had been vaccinated, one patient had missed an appointment for vaccination and rescheduled a new appointment in the following weeks, and the third patient could not be reached for further discussion. Of the 17 patients who engaged in tailored conversations and expressed concerns regarding vaccine safety and/or efficacy, three received vaccination 30 days later, four decided against vaccination and ten continued to think about it.	The quality improvement project showed that dialogue-based interventions that are conducted by telephone and are tailored to the specific vaccination barriers faced by older adults may have some effectiveness in encouraging vaccination against COVID-19.
Ghadieh et al., 2015, Lebanon (Beirut) [33]	Using different types of reminder inviting individuals to get the pneumococcal vaccine. Six equal subgroups were included. Subgroups 1a, 2a, and 3a received, respectively, a standardized phone call reminder by a nurse, a SMS-text reminder, and an e-mail reminder. Subgroups 1b, 2b, and 3b also received an additional brief educational intervention about the seriousness of pneumococcal disease. Each subgroup received three identical reminders given at four-week intervals. Patients were asked to call the clinic themselves and schedule an appointment to receive the vaccine.	The rate of vaccination increased from 17.2% to 20.4% before and after this intervention in patients older than 65 years of age.	Use of electronic reminders via e-mail and mobile phones seems to be a feasible and sustainable method to increase pneumococcal vaccination rates in primary care centers.
Hull et al., 2002, UK (East London and Essex) [34]	Telephone calls from the practice receptionist to intervention group offering an appointment for influenza immunization at a nurse-run clinic. Follow-up period: 2 months.	Adjusted* differences in immunization rate between control and intervention groups: 6.3%, 95% CI [0.7–12.0], *p* = 0.026. OR of intervention on rate of immunization = 1.29 *, 95% CI [1.03–1.62], *p* = 0.026 *. * adjusted for clustering within clinics and households.	General practices can boost immunization rates for influenza vaccination among the fit older population by about 6% using telephone calls from practice receptionists. This effect was achieved in addition to national and community advertising and mail campaigns.
Humiston et al., 2011, United States (Rochester) [35]	The intervention group received a staged intervention of provider reminders (patient charts were flagged with a reminder for the physician indicating that the patient was not vaccinated), patient recall (mailed influenza immunization reminders in the form of a letter or card), and patient outreach (phone calls to those that had no routine appointment scheduled during the 3-month flu-vaccine period to ask them to make an appointment. If the patient did not make an appointment within two weeks, another call was attempted). Patients in the control group received routine care. Influenza immunization coverage was measured prior to enrollment and on the end date.	Immunization rates were greater for the intervention group than for the control group (64% vs. 22%, *p* = 0.0001). Adjusted logistic regression analysis showed that patients in the intervention group were more than six times as likely to be vaccinated than patients in the control group: (OR = 6.27, 95% CI [5.42–7.26], *p* = 0.0001)	Patient tracking/recall/outreach and provider prompts were intensive but successful approaches to increasing seasonal influenza immunization rates among this group of inner-city seniors.
Kellerman et al., 2000, United States (Kansas) [36]	All 475 patients of a family practice center received a postcard urging prompt influenza immunization. Those not responding within 1 month were systematically allocated either to a group receiving further telephone contact or to a control group (postcard only). A maximum of 2 telephone calls were made, the first occurring as closely as possible to 1 month following the postcard intervention, the second occurring after several more days, if the first phone call was unsuccessful.	No additional immunizations could be attributed to the telephone intervention.	No significant increase in immunization rates among patients receiving an additional telephone intervention, compared with patients receiving postcard reminders only.
Klassing et al., 2017, United States (Kansas City) [37]	Patients were randomized into one of three study arms: a phone call, a mailed letter, or no intervention (control). A phone call script was utilized in the call group, while the letter group received a standardized letter addressed to each specific patient. All subjects were exposed to in-store advertising for the seasonal influenza vaccine and received flyers advertising on-site immunizations when picking up prescriptions during the study period. After the intervention, a review of electronic pharmacy vaccination records and consent forms was performed to determine vaccination rates within the study groups. If no documentation of vaccination was found, one follow-up phone call was made to determine if the patient received an influenza or pneumococcal vaccination at a non-study pharmacy, clinic, or other location.	Sub-analysis was conducted for patients with asthma and/or COPD over the age of 65, but no significant difference in vaccine rates was found between the interventions and the control groups.	For individuals 65 years and over, both the phone call and the letter interventions did not show a significant increase in immunization rates.
Krieger et al., 2000, United States (Washington) [38]	The intervention group received educational brochures mailed with a postage-paid reply card to report immunization status. If no reply card was received or if the card indicated that immunization was lacking, a volunteer called the participant using a script to encourage receipt of immunizations and to address specific barriers to immunization. They also had a follow-up contact to ascertain whether immunization(s) were received (self-reported). The control group received standard senior center and community immunization promotion activities (e.g., newsletter articles, health fairs).	Among participants without prior pneumococcal immunization, the pneumococcal immunization rate among the intervention group (52.0%, 95% CI [46.6–57.4]) was significantly higher than that of the control group (30.9%, 95% CI [26.6–35.2]) (Rate Ratio = 1.68, 95% CI [1.40–2.03]). Among those without influenza immunization in the prior year, significantly more (50.0%, 95% CI [40.0–60.0]) were immunized against influenza in the intervention group than in the control group (23.0%, 95% CI [15.2–33.3]) (Rate Ratio 2.17, 95% CI [1.42–3.31]). Among those with influenza immunization in the prior year, the Rate Ratio was 1.04 (95% CI [1.01–1.07]).	The intervention increased both influenza and pneumococcal immunization rates to high levels, suggesting that further progress in increasing adult immunization coverage is possible.
Minor et al., 2010, United States [39]	The mail reminder group received a letter from the clinic via mail and a copy of the CDC influenza vaccine information statement. The phone reminder group received a personal phone call from a doctor or pharmacy resident within the clinic. Both groups received the same information regarding the influenza vaccination, including explanations of the importance of vaccination and general indications. The control group received standard care. The following spring, records for all included patients were screened for documentation of influenza vaccination. Those without documentation received a phone call from the same person inquiring about their vaccination status and approximate date, if vaccination was received at another site.	Observed vaccination rates in individuals > 65 years old: Females: control group: 50.0%, mail: 54.4%, phone: 58.9%. Males: control group: 58.3%, mail: 58.3%, phone: 87.5%.	Both phone and mail reminders were more effective than the control. Phone reminders resulted in higher vaccination rates with a better response in all age/sex groups.
Strain et al., 2021, Canada (Alberta) [40]	A brief discussion on influenza vaccination between pharmacy staff and unvaccinated individuals > 65 years of age. Primary outcome: to have an appointment for influenza vaccine administration arranged by the end of the telephone consultation. As a secondary process evaluation outcome, the impact of information contained in these scripted conversations were evaluated, in order to increase vaccine options for those > 65 years of age.	A total of 643 individuals did not have a documented influenza vaccine from their primary provider. Of them, 169 (26.3%) had been vaccinated in another setting. Of the remaining 474, a total of 316 (67%) agreed to receive the vaccine by the end of the telephone consultation.	A short and practical pharmacy intervention in three Canadian provinces was able to reach the WHO 75% influenza immunization target for older adults.
Winston et al., 2007, United States (Atlanta) [41]	A telephone call initiated by a nurse informed patients that pneumococcal vaccination was recommended and was a covered benefit of their insurance. The primary outcome was a 6-month follow-up for pneumococcal vaccination, while secondary outcomes investigated the proportion and characteristics of patients in the intervention arm who reported that they were already vaccinated (the primary reason for other patients to choose not to vaccinate), and vaccination interest and uptake among intervention arm patients reached by telephone who confirmed that they had not been previously vaccinated.	Vaccination status for the elderly group at 6 months after randomization: 17% (201/1198) of intervention patients were vaccinated versus 8% (100/1197) of controls (*p* < 0.001).	The telephone intervention was successful at increasing vaccination rates in a diverse managed care population that had already received mailed reminders.
TEXT MESSAGES			
Esteban-Vasallo et al., 2019, Spain (Madrid) [42]	SMS reminders were sent to patients with a delayed vaccination status, inviting them to take an appointment and get a flu vaccination.	Receiving the reminder was associated with a significantly higher probability of vaccination for the over-65 population that had at least one concurrent chronic condition in addition to their rare disease (IRR = 1.23, 95% CI [1.08–1.40]). When analyzing by sex: men with at least a concurrent chronic condition in addition to their rare disease (IRR: 1.58, 95% CI [1.25–2.00]) and for woman without any concurrent chronic conditions (IRR: 1.40, 95% CI [1.05–1.89]).	Although the intervention was modestly effective, it proved beneficial in some cases. It can be an additional strategy to improve vaccine uptake, since it is simple, feasible, affordable, and easily scalable, particularly when immunization and target population data are available in population registries.
Patel et al., 2022, United States (Northeastern) [43]	Nineteen different text messaging protocols were generated by behavioral scientists. Protocols varied in their contents and/or timing of up to two sets of text reminders. Reminders to get a flu shot were sent from the patient’s healthcare provider in the 3 days preceding the patient’s appointment.	For patients aged 65 years and older, none of the 19 nudge protocols were significantly effective in increasing vaccination rates compared with controls, although 8 of the 19 nudge protocols were effective among the entire population and subgroup analysis showed that there were no significant differences between age groups.	Nudges were not effective in boosting vaccination rates among older adults.
Regan et al., 2017, Australia [18]	Half of the patients within each practice were randomly assigned to receive a SMS (intervention group) or no SMS (control group), The SMS reminded patients of their eligibility for a free influenza vaccine and invited them to call their practice to schedule an appointment. General practice staff were blinded to the patient’s group assignment. After the intervention, secondary data extraction from participating patient electronic medical records was used to identify the date of administration for the influenza vaccination received.	For patients 65 years of age or older, 20.5% (n = 376) of the intervention group and 15.8% (n = 281) of the control group were vaccinated during the study period. (RR = 1.26, 95% CI [1.10–1.45], *p* < 0.05).	SMS reminders for seasonal influenza vaccination significantly increased the proportion of high-risk patients who received the vaccine.
Tubiana et al., 2021, France and Monaco [44]	The intervention arm received a multifaceted intervention at the end of an ED visit, including (a) a brief structured interview about pneumococcal and influenza burdens and the interest of both vaccinations, (b) an information sheet, (3) a letter to their GP stating that the patient was at risk for pneumococcal infection and could benefit from pneumococcal vaccination, (4) 3 text message reminders sent every 2 weeks. Patients randomized into the control arm received the same intervention, except they did not receive any text message and had a non-structured interview about pneumococcal risk and vaccination. The primary outcome was self-reported pneumococcal vaccination within 6 months of enrollment. Secondary outcomes included 6-month self-reported influenza vaccination and 12-month all-cause mortality.	In the intention-to-treat analysis, the multifaceted intervention did not alter the pneumococcal vaccination rate: 6.4% versus 4.6%, 95% CI [−0.9–4.4], *p* = 0.19. On the other hand, the intervention improved influenza vaccination rates: 52.1% versus 40.0%, 95% CI [2.4–21.8], *p* = 0.01.	A multifaceted intervention based on text message reminders provides an opportunity to increase influenza vaccination among elderly patients visiting an ED.
ELECTRONIC MESSAGES SENT VIA PERSONAL HEALTH RECORD			
Szilagyi et al., 2020, United States (Los Angeles) [45]	Patients due for an influenza vaccine were sent a letter via the patient portal of their health care system, which included educational information (i.e., regarding the importance and safety of influenza vaccines), a recommendation to make an appointment to get the vaccine, and a website link to input influenza vaccinations received elsewhere. Patients were randomized within primary care practices into 1 of 4 groups (no reminder, 1 reminder, 2 reminders, or 3 reminders). Portal reminders were sent at the beginning of October, November, and December (depending on the study group). The electronic health record documented any influenza vaccines and merged data from external sources (e.g., pharmacies). The primary analysis excluded vaccinations reported only by patients in response to the portal reminders because the control group did not have this opportunity to self-report, thus eliminating differential outcome ascertainment. Secondary outcomes were influenza vaccination rates among the subgroups and influenza vaccinations received elsewhere (which were self-reported by patients in reply to the portal-based query).	For patients 65 years or older, the following was reported. Influenza vaccination rates excluding self-reported vaccinations: no statistically significant effect (vaccination rates: 53.2% in the control group, 53.1% in the 1-reminder group, 53.0% in the 2-reminder group, and 53.8% in the 3-reminder group, *p* = 0.31). Influenza vaccination rates including self-reported vaccinations: portal reminders were effective (vaccination rates: 53.6% in the control group, 54.6% in the 1-reminder group, 55.1% in the 2-reminder group, and 56.7% in the 3-reminder group, *p* < 0.001).	When excluding self-reported vaccination, the intervention had no effect on vaccination rates among older adults. Statistically significant improvements in vaccination rates were noted for the elderly population when also considering self-reported vaccinations.
Szilagyi et al., 2021, United States (Los Angeles) [46]	Electronic messages were sent via EHR incorporating the following behavioral science strategies: (1) pre-commitment (to their doctor that they will obtain the influenza vaccine, a strategy in which people are asked to commit today to engage in a future target behavior), and (2) gain/loss framing (a strategy in which a message is described as what a person has to gain or lose by taking a particular action). Patients were randomized into either (1) pre-commitment reminder alone, (2) pre-commitment + loss frame messages, (3) pre-commitment + gain frame messages, (4) loss frame messages alone, (5) gain frame messages alone, or (6) standard care control. Patients in the precommitment group were sent a message in mid-October, asking if they planned on getting an influenza vaccine. Patients in the loss or gain frame groups were sent up to 3 portal reminders (late October, November, and December, if no documented influenza vaccination was found in their EHR) about the importance and safety of influenza vaccines.	For patients 65 years or older, and excluding vaccinations self-reported by patients in response to the reminders, influenza vaccination rates by pre-commitment and by reminder framing were not statistically different. Multivariate analyses (both adjusted and non-adjusted) show no statistically significant impact of either pre-commitment or loss/gain framing on influenza vaccination rates. Including self-reported vaccination in response to portal reminders, influenza vaccination rates by pre-commitment and by reminder framing were not statistically different. Adjusted multivariate analyses comparing loss/gain frame and pre-commitment (no/yes) showed a small statistically significant effect of loss framing: RR = 1.03, 95% CI [1.01–1.05], *p* < 0.01.	Influenza vaccination rates were not statistically different for any of the study groups vs. control. When incorporating self-reported vaccinations, adjusted multivariate analyses showed a small statistically significant effect of the loss framing strategy.
Otsuka et al., 2013, United States (Ohio State University) [19]	Patients in the intervention groups received an informational packet regarding shingles and the herpes zoster vaccine through their electronic medical record or through the US postal service on the basis of their personal health record status. Patients were instructed to contact the clinic if they were interested in receiving the herpes zoster vaccine or to have their medical record updated if they had already received the vaccine. If indicated, a prescription for the herpes zoster vaccine was mailed to the patient with instructions on how to obtain it. Six months after the intervention, a second electronic medical record report was generated to determine the change in vaccination rate of both the intervention and control groups.	In the personal health record population, 13.2% (n = 33/250) of the intervention group had a documented vaccination compared with 5.0% (n = 21/424) of controls (relative risk, 2.7, 95% CI [1.6–4.5], *p* = 0.0001). In the non-personal health record population, the vaccination rates were 5.2% (n = 13/250) in the intervention group and 1.8% (n = 30/1665) in the control group (relative risk, 2.9, 95% CI [1.6–5.5], *p* = 0.0007) The outcome of the logistic regression interaction likelihood ratio test revealed that the 2 intervention effects did not differ significantly (*p* = 0.99).	Communication outside of face-to-face office visits, by both personal health record electronic messages and information by mail, can improve preventative health intervention rates compared with standard care.
AUTOMATED PHONE CALLS			
Bedwick et al., 2017, United States (Morgantown) [47]	A prerecorded phone message was sent to all eligible patients over a period of 1 week at the beginning of the study and monthly thereafter for a total of 3 phone calls per patient spanning 3 months. At the completion of the study, the total number of herpes zoster immunizations given during the study period was compared with the number given during the same period of the previous year.	The telephone message was sent to approximately 600 patients, and a total of 25 herpes zoster vaccines were given during the intervention period, compared with 16 during the control period.	An automated and targeted telephone call directed at eligible patients may lead to an increase in vaccination numbers.
Hurley et al., 2018, United States (Denver) [48]	Individuals randomized into the intervention arm received up to two auto-dial phone calls followed by a postcard prompting them to receive vaccines. Documentation of necessary vaccinations in the immunization information system < 6 months after the reminder/recall was the primary outcome. The control arm received standard care that did not include any reminders to receive vaccines.	For the 65-years-and-over population, 32.0% (n = 847) of the intervention group received the influenza vaccine versus 28.6% (n = 760) in the control group (*p* < 0.01). No significant difference was found for the pneumococcus vaccine.	Centralized reminders/recalls were effective at increasing influenza vaccination rates in adults aged >65 years over a short time period, without a large burden to the practices, and at a reasonable cost. No significant effect was found for pneumococcal vaccination rates.
Stolpe et al., 2019, United States (Northeastern) [49]	A total of 22,301 patients with an identified vaccination gap were randomly assigned in a 1:1 ratio to intervention or control. An automated telephone call offering the vaccines was made to the intervention patients and asked to give a vocal response indicating the intent to receive the vaccine during their next visit to the pharmacy. Patients in the control group received a scheduled outbound communication without the added vaccination prompt. The primary outcome was the proportion with administration of at least 1 of the vaccines between March 2015 and January 2016.	The intervention did not significantly increase the vaccination rates. The subgroup logistic regression analysis showed no significant effect also for individuals aged 60 years and older (unadjusted OR = 1.01 [0.84–1.24] adjusted OR = 1.02 [0.84–1.23]).	The automated phone call-based intervention did not significantly increase adult vaccination rates. The relatively low call completion rate is indicative of inefficiencies in the modality overall.
REMOTE PATIENT MONITORING IN A HOME TELEHEALTH PROGRAM			
Rand et al., 2022, United States (San Francisco) [50]	Automated 2-way messaging offering the influenza vaccination was transmitted during September and October 2020 using remote patient monitoring. Patients were prompted to respond to a question asking whether they had received a flu vaccine anywhere else. In January 2021, missing information was reconciled and tailored education was provided during a telephone visit for patients that had no vaccination plans. Patients’ electronic health records were examined over 2 flu seasons. During the 2019 to 2020 flu season, Home Telehealth patients received the usual care provided by outpatient clinic teams, which incorporated vaccination administration during face-to-face visits. During the 2020 to 2021 flu season, Home Telehealth patients received the usual care and the new intervention.	Of veterans 66 years and older, 81.7% received the vaccine during the 2020–2021 flu season, but the intervention was insignificant in increasing vaccination rates compared to the previous flu season.	The introduction of the clinical intervention, incorporating tailored education to encourage vaccinations, was insignificant in increasing rates. Patients who declined vaccinations pre-intervention continued to decline during the 2020–2021 flu season.

## Data Availability

No new data were created or analyzed in this study. Data sharing is not applicable to this article.

## Appendix A

### Appendix A.1. Search Strategy: Keywords and Limits

Axis 1 Keywords
influenza vaccin*; influenza immuni*; herpes zoster vaccin*; herpes zoster immuni*; shingles vaccin*; shingles immuni*; covid* vaccin*; covid* immuni*; pneumococc* vaccin*; pneumococc* immuni*
Axis 2 Keywords
text messag*; cell phone messag*; mobile phone messag*; cell phone; telephone; SMS messag*; MMS messag*; information technology; email*; application*; phone call*; reminder systems; social network*; social media; communications media; reminder*; recall*
Axis 3 Keywords
aged; elderly; older adult*; senior
Axis 4 Keywords
vaccination; vaccin* uptake; vaccin* coverage; vaccin* rate*; immunisation coverage; immunisation rate; vaccination hesitancy; vaccination refusal
Limits
The search strategy was limited to papers published between 1 January 2000, and 10 November 2022, human study populations and English language

### Appendix A.2. Search Strings Definition, Pubmed Database

((((text messag*[Title/Abstract]) OR (“Text Messaging”[Mesh]) OR (“Communications Media”[Mesh]) OR (cell phone messag*[Title/Abstract]) OR (“Cell Phone”[Mesh]) OR (mobile phone messag*[Title/Abstract]) OR (SMS messag*[Title/Abstract]) OR (“Reminder Systems”[Mesh]) OR (“Telephone”[Mesh]) OR (MMS messag*[Title/Abstract]) OR (information technology[Title/Abstract]) OR (email*[Title/Abstract]) OR (application*[Title/Abstract]) OR (phone call*[Title/Abstract]) OR (social network*[Title/Abstract]) OR (social media[Title/Abstract]) OR (reminder*[Title/Abstract]) OR (recall*[Title/Abstract])) AND ((elderly) OR (“Aged”[Mesh]) OR (older adult*) OR (senior)) AND ((influenza vaccin*[Title/Abstract]) OR (influenza immuni*[Title/Abstract]) OR (herpes zoster vaccin*[Title/Abstract]) OR (herpes zoster immuni*[Title/Abstract]) OR (shingles vaccin*[Title/Abstract]) OR (shingles immuni*[Title/Abstract]) OR (covid* vaccin*[Title/Abstract]) OR (covid* immuni*[Title/Abstract]) OR (pneumococc* vaccin*[Title/Abstract]) OR (pneumococc* immuni*[Title/Abstract])) AND ((“Vaccination”[Mesh]) OR (“Vaccination Coverage”[Mesh]) OR (“Vaccination Hesitancy”[Mesh]) OR (“Vaccination Refusal”[Mesh]) OR (vaccin* uptake[Title/Abstract]) OR (vaccin* coverage[Title/Abstract]) OR (vaccin* rate*[Title/Abstract]) OR (immunisation coverage[Title/Abstract]) OR (immunisation rate[Title/Abstract]))) AND ((“2000/01/01”[Date—Publication]: “2022/11/10”[Date—Publication]))) AND (“English”[Language])

### Appendix A.3. Search Strings Definition, Scopus Dastabase

(TITLE-ABS (text AND messag*) OR TITLE-ABS (cell AND phone AND messag*) OR TITLE-ABS (mobile AND phone AND messag*) OR TITLE-ABS (sms AND messag*) OR TITLE-ABS (mms AND messag*) OR TITLE-ABS (information AND technology) OR TITLE-ABS (email*) OR TITLE-ABS (application*) OR TITLE-ABS (phone AND call*) OR TITLE-ABS (social AND network*) OR TITLE-ABS (social AND media) OR TITLE-ABS (reminder*) OR TITLE-ABS (recall*)) AND (ALL (elderly) OR ALL (older AND adult*) OR ALL (senior)) AND (TITLE-ABS (influenza AND vaccin*) OR TITLE-ABS (influenza AND immuni*) OR TITLE-ABS (herpes AND zoster AND vaccin*) OR TITLE-ABS (herpes AND zoster AND immuni*) OR TITLE-ABS (shingles AND vaccin*) OR TITLE-ABS (shingles AND immuni*) OR TITLE-ABS (covid* AND vaccin*) OR TITLE-ABS (covid* AND immuni*) OR TITLE-ABS (pneumococc* AND vaccin*) OR TITLE-ABS (pneumococc* AND immuni*)) AND (TITLE-ABS (vaccin* AND uptake) OR TITLE-ABS (vaccin* AND coverage) OR TITLE-ABS (vaccin* AND rate*) OR TITLE-ABS (immunisation AND coverage) OR TITLE-ABS (immunisation AND rate)) AND (LIMIT-TO (PUBYEAR, 2022) OR LIMIT-TO (PUBYEAR, 2021) OR LIMIT-TO (PUBYEAR, 2020) OR LIMIT-TO (PUBYEAR, 2019) OR LIMIT-TO (PUBYEAR, 2018) OR LIMIT-TO (PUBYEAR, 2017) OR LIMIT-TO (PUBYEAR, 2016) OR LIMIT-TO (PUBYEAR, 2015) OR LIMIT-TO (PUBYEAR, 2014) OR LIMIT-TO (PUBYEAR, 2013) OR LIMIT-TO (PUBYEAR, 2012) OR LIMIT-TO (PUBYEAR, 2011) OR LIMIT-TO (PUBYEAR, 2010) OR LIMIT-TO (PUBYEAR, 2009) OR LIMIT-TO (PUBYEAR, 2008) OR LIMIT-TO (PUBYEAR, 2007) OR LIMIT-TO (PUBYEAR, 2006) OR LIMIT-TO (PUBYEAR, 2005) OR LIMIT-TO (PUBYEAR, 2004) OR LIMIT-TO (PUBYEAR, 2003) OR LIMIT-TO (PUBYEAR, 2002) OR LIMIT-TO (PUBYEAR, 2001) OR LIMIT-TO (PUBYEAR, 2000)) AND (LIMIT-TO (DOCTYPE, “ar”)) AND (LIMIT-TO (LANGUAGE, “English”)) AND (EXCLUDE (SUBJAREA, “VETE”))

## Appendix B

**Table A1:** Quality assessment of the included studies. Cochrane Risk of Bias 2.0 (RoB 2) Tool for Randomized Controlled Trials.

Study (Main Author, Year)	Randomization Process	Deviations from Intended Interventions	Missing Outcome Data	Measurement of the Outcome	Selection of the Reported Result	Overall Bias
Ghadieh et al., 2015 [33]	Low	Low	Low	Low	Low	Low
Hull et al., 2002 [34]	Low	Low	Low	Low	Low	Low
Humiston et al., 2011 [35]	Low	Low	Low	Low	Low	Low
Hurley et al., 2018 [48]	Low	Low	Low	Low	Low	Low
Klassing et al., 2017 [37]	Low	Low	Low	Low	Low	Low
Krieger et al., 2000 [38]	Low	Low	Low	Low	Low	Low
Patel et al., 2022 [43]	Low	Low	Low	Low	Low	Low
Minor et al., 2010 [39]	Some concerns	Low	High	Low	Low	High
Otsuka et al., 2013 [19]	Some concerns	Low	Low	Low	Low	Some concerns
Regan et al., 2017 [18]	Low	Low	Low	Low	Low	Low
Stolpe et al., 2019 [49]	Low	Low	Low	Low	Low	Low
Szilagyi et al., 2020 [45]	Low	Low	Low	Low	Low	Low
Szilagyi et al., 2021 [46]	Low	Low	Low	Low	Low	Low
Tubiana et al., 2021 [44]	Low	Low	Low	Low	Low	Low
Winston et al., 2007 [41]	Low	Low	Low	Low	Low	Low

**Table A2:** Quality assessment of the included studies. Risk of Bias in Non-randomized Studies—of Interventions (ROBINS-I) for Non-Randomized Trials.

Study (Main Author, Year)	Bias due to Confounding	Bias in Selection of Participants into the Study	Bias in Classification of Interventions	Bias due to Deviations from Intended Interventions	Bias due to Missing Data	Bias in Measurement of Outcomes	Bias in Selection of the Reported Result	Overall Bias
Biyik et al., 2020 [31]	Serious	Low	Low	Low	Low	Low	Low	Moderate
Esteban-Vasallo et al., 2019 [42]	Low	Serious	Serious	Serious	Low	Low	Moderate	Serious
Kellerman et al., 2000 [36]	Moderate	Low	Low	Low	Low	Low	Low	Low

**Table A3:** Quality assessment of the included studies. National Institutes of Health (NIH) Quality Assessment Tool for before–after (Pre–Post) Study with no Control Group.

Study (Main Author, Year)	Major Components												Quality Rating
	1	2	3	4	5	6	7	8	9	10	11	10	
Bedwick et al., 2017 [47]	Yes	Yes	Yes	Yes	Yes	Yes	Yes	No	N.A.	No	No	No	Fair
Desir et al., 2022 [32]	Yes	Yes	Yes	No	No	Yes	Yes	No	Yes	No	No	N.A.	Poor
Rand et al., 2022 [50]	Yes	Yes	Yes	No	Yes	Yes	No	No	Yes	No	No	No	Poor
Strain et al., 2021 [40]	Yes	Yes	Yes	Yes	Yes	Yes	Yes	No	Yes	No	No	N.A.	Fair

N.A. = Not Applicable/Cannot Determine/Not Reported.
